# Mitochondrial Protein SLIRP Affects Biosynthesis of Cytochrome c Oxidase Subunits in HEK293T Cells

**DOI:** 10.3390/ijms25010093

**Published:** 2023-12-20

**Authors:** Mariia V. Baleva, Uliana Piunova, Ivan Chicherin, Ruslan Vasilev, Sergey Levitskii, Piotr Kamenski

**Affiliations:** Faculty of Biology, Lomonosov Moscow State University, 1/12 Leninskie Gory, 119234 Moscow, Russia; mary-bw@mail.ru (M.V.B.); ulya.ulichka@gmail.com (U.P.); i.v.chicherin@gmail.com (I.C.); ruavasilev@gmail.com (R.V.)

**Keywords:** mitochondria, translation, translation regulation

## Abstract

Mitochondria carry out various vital roles in eukaryotic cells, including ATP energy synthesis, the regulation of apoptosis, Fe-S cluster formation, and the metabolism of fatty acids, amino acids, and nucleotides. Throughout evolution, mitochondria lost most of their ancestor’s genome but kept the replication, transcription, and translation machinery. Protein biosynthesis in mitochondria is specialized in the production of highly hydrophobic proteins encoded by mitochondria. These proteins are components of oxidative phosphorylation chain complexes. The coordination of protein synthesis must be precise to ensure the correct assembly of nuclear-encoded subunits for these complexes. However, the regulatory mechanisms of mitochondrial translation in human cells are not yet fully understood. In this study, we examined the contribution of the SLIRP protein in regulating protein biosynthesis in mitochondria. Using a click-chemistry approach, we discovered that deletion of the *SLIRP* gene disturbs mitochondrial translation, leading to the dysfunction of complexes I and IV, but it has no significant effect on complexes III and V. We have shown that this protein interacts only with the small subunit of the mitochondrial ribosome, which may indicate its involvement in the regulation of the mitochondrial translation initiation stage.

## 1. Introduction

According to the widely accepted endosymbiotic hypothesis, mitochondria derived from an ancient alpha-proteobacterium [1] through endosymbiosis and subsequently evolved into a vital constituent of the eukaryotic cell. Mitochondria serve not only as an energy generator for maintaining the cellular homeostasis but also actively participate in the biogenesis of various macromolecules like lipids, heme and iron–sulfur clusters. Mitochondria, in contrast to most other organelles, possess their own genome which was inherited from their ancestral bacteria, albeit in a considerably reduced form [2]. For instance, the DNA of free-living bacteria encodes 1500–7500 genes, whereas human mitochondrial DNA encodes only 13 proteins. Mitochondria have a translation system consisting of mitoribosomes and several protein factors that regulate protein synthesis. Mitochondrially encoded proteins in human cells are synthesized from nine monocistronic and two bicistronic messenger RNAs (mRNAs). All of these mRNAs lack a leader sequence; i.e., their 5′ untranslated regions (UTRs) are either limited to a few nucleotides or are completely absent. In bacteria, the 70S ribosome triggers the translation process on leaderless mRNAs with start codons at their 5′ end [3]. As mitochondria originate from bacteria, it may be inferred that the 55S monosome utilizes a comparable mechanism to initiate translation on mammalian leaderless mRNAs. This assumption is supported by the results of various in vitro studies [4,5]. The recruitment of mRNA by the mitochondrial ribosome’s mechanism is still unknown. Some in vitro studies have demonstrated that the mitochondrial 55S ribosome has a greater affinity for the first AUG codon [4,6] although some mitochondrial mRNAs start with non-canonical codons [7]. Due to the lack of steady secondary structures at their 5′ ends, mitochondrial mRNAs can pass through the ribosomal entry channel [8]. Additionally, extensions of the uS5m protein specific to mitochondria, which are abundant in amino acids carrying a positive charge, constitute the channel responsible for guiding mRNA with a negative charge to the P-site. At this site, the mRNA is secured by a codon–anticodon interaction [9]. Structural studies have revealed that the protein mS39 (PTCD3), belonging to the large PPR family of RNA-binding proteins, is located at the entrance of the ribosome channel and may be involved in mRNA recruitment by binding the U-rich sequence starting after codon 7 [9,10]. Mutations within the gene of mS39 have been observed to cause mitochondrial respiration dysfunction as a result of insufficiency of complexes I and IV of the respiratory chain [11,12]. Cryo-electron microscopy data, based on mass spectrometry, indicate that the mS39 protein in the 55S ribosome interacts with the LRPPRC–SLIRP complex. Like mS39, the LRPPRC protein belongs to PPR proteins and has several contacts with mitochondrial transcripts [13]. No convincing evidence supports the participation of the LRPPRC–SLIRP complex in delivering mRNA to the entry channel of the mitochondrial ribosome. However, the deletion of LRPPRC disrupts the translation of certain mRNAs, including ND3, ND6, ATP6, and CO I mRNA [14]. SLIRP, an RNA-binding protein that interacts with SRA stem loops, regulates nuclear receptors by binding to steroid receptor RNA activator (SRA RNA) through its RRM domain [15]. However, the majority of SLIRP is directed toward mitochondria through the MTS signal. While the exact mitochondrial function of SLIRP remains unclear, it is known to contribute to the stability of mitochondrial transcripts [16] and may play a role in the process of mitochondrial translation [9]. Nonetheless, the involvement of the SLIRP protein in mitochondrial translation has not yet been sufficiently investigated.

To clarify the role of SLIRP in the mitochondrial translation apparatus, we generated the HEK293T cell line with a deletion in the *SLIRP* gene, resulting in the absence of synthesis of the functional protein. The loss of SLIRP function in the cells of the resulting line led to a reduction in mitochondrial respiration, which was caused by a decrease in the levels of respiratory chain complexes I and IV. This was associated with a reduction in the translation of certain mitochondrial transcripts corresponding to the complexes, in particular CO I, CO II and CO III. In contrast, the quantities of complexes III and V and the translation of their mitochondrially encoded subunits remained unchanged. Moreover, we demonstrated that SLIRP associates exclusively with small subunits of the mitochondrial ribosome in sucrose density gradients. Taken together, these data allow us to suppose the selective action of SLIRP in mitochondrial translation regulation.

## 2. Results

### 2.1. Knockout of SLIRP Gene Impairs Mitochondrial Fitness and Complex I and IV Activities

**Figure 1:**
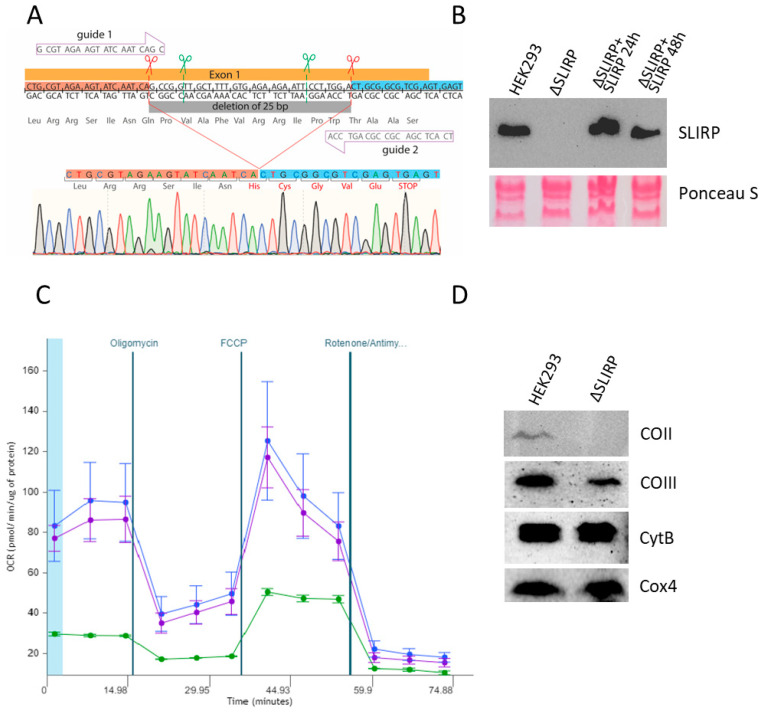
Deletion in the *SLIRP* gene leads to mitochondrial dysfunction and decrease in mitochondrially encoded complex IV subunits. (**A**) Scheme of the *SLIRP* gene deletion, sgRNA located on the gene sequence, green scissors indicate planned breaks, red scissors—true deletion confirmed by Sanger sequencing. (**B**) Western blot analysis of cell lysates from wild-type cells, knockout cells and knockout cells transfected with SLIRP-containing plasmid after 24 and 48 h of cultivation. (**C**) Diagram of oxygen consumption rate under normal conditions and after addition of oligomycin, FCCP and rotenone/antimycin A (indicated above) obtained by the Seahorse XF HS Mini. The blue line represents wild-type cells, the green line represents SLIRP knockout cells, and the *pink line* represents SLIRP knockout cells transfected with SLIRP cDNA-containing plasmid for 24 h. Data represented as mean values of 3 biological replicates +/− SD. (**D**) Western blot analysis of complex III and IV subunits in wild-type and SLIRP knockout cells. Antibodies to nuclear encoded Cox4 protein were used as a loading control.

SLIRP was initially identified as a protein that binds SRA RNA and regulates nuclear receptor activity. However, over 90% of the protein is located in mitochondria [15]. Clinical studies have demonstrated that mutations in the *SLIRP* gene cause mitochondrial encephalomyopathy [17], thus highlighting the crucial role of the SLIRP protein in maintaining proper mitochondrial function. To determine the function of the SLIRP protein in mitochondria, we generated the HEK293T cell line with a deletion of 32 nucleotides in the first exon of the *SLIRP* gene (Figure 1A). The editing of the genome was performed using CRISPR/Cas9 technology with two single guide RNAs. After sorting and cloning of the cells, we identified three clones with confirmation of the deletion by PCR. Sanger sequencing revealed that the deletion caused a reading frame shift, resulting in a stop-codon after five amino acid residues. The absence of full-length protein in the knockout cell line was confirmed by Western blot analysis. The levels of SLIRP protein were recovered to wild-type levels 24 and 48 h after the transfection of knockout cells with the plasmid carrying SLIRP cDNA (Figure 1B).

Next, we aimed to assess the influence of the SLIRP protein on mitochondrial function. To achieve this objective, we utilized the Seahorse XF HS MINI analyzer to quantify oxygen consumption rates in cells where the *SLIRP* gene was deleted and wild-type cells under the influence of the complex V inhibitor oligomycin, the uncoupler carbonyl cyanide 4-(trifluoromethoxy)phenylhydrazone (FCCP), and inhibitors of complexes I and III—rotenone and antimycin A. The experiments showed that the absence of the SLIRP protein leads to a significant decrease in mitochondrial respiration, and the absence of a response to the uncoupler (FCCP) indicates the dysfunction of complex IV. Importantly, the expression of the *SLIRP* gene in knockout cells for over 48 h restored the metabolic phenotype. These findings highlight the critical involvement of SLIRP absence in mitochondrial functioning (Figure 1C).

We also calculated the coupled ATP production rates in HEK293T and SLIRP knockout cells (Figure S1). The mitochondrial ATP production in SLIRP knockout cells was more than five times lower than in wild-type cells, indicating significant mitochondrial dysfunction. These data were consistent with those obtained when the proliferative activities of wild-type and SLIRP knockout cells were examined. When cultured in high-glucose medium, knockout cells lagged significantly behind wild-type cells in the early stages (72–96 h) but practically reached wild-type growth during long-term cultivation (120 h or more, Figure S2). In contrast, knockout cells lagged significantly behind wild-type cells throughout the experiment when cultivated in galactose-containing medium (Figure S2). These data suggest that knockout cells were highly dependent on glycolysis due to impaired mitochondrial function.

To investigate whether the reduced activity of complex IV resulted from a decrease in the protein levels of the mitochondrially encoded subunits, we performed Western blot analysis. Our data showed that the levels of the mitochondrially encoded CO II and CO III subunits were significantly reduced in the knockout cells. In contrast, the levels of nuclear-encoded complex IV subunit Cox4 and mitochondrial-encoded complex III subunit CytB were similar in knockout and wild-type cells (Figure 1D).

### 2.2. The Absence of SLIRP Leads to Deficiency in OXPHOS Complexes I and IV Caused by Imbalance in Mitochondrial Protein Synthesis

**Figure 2:**
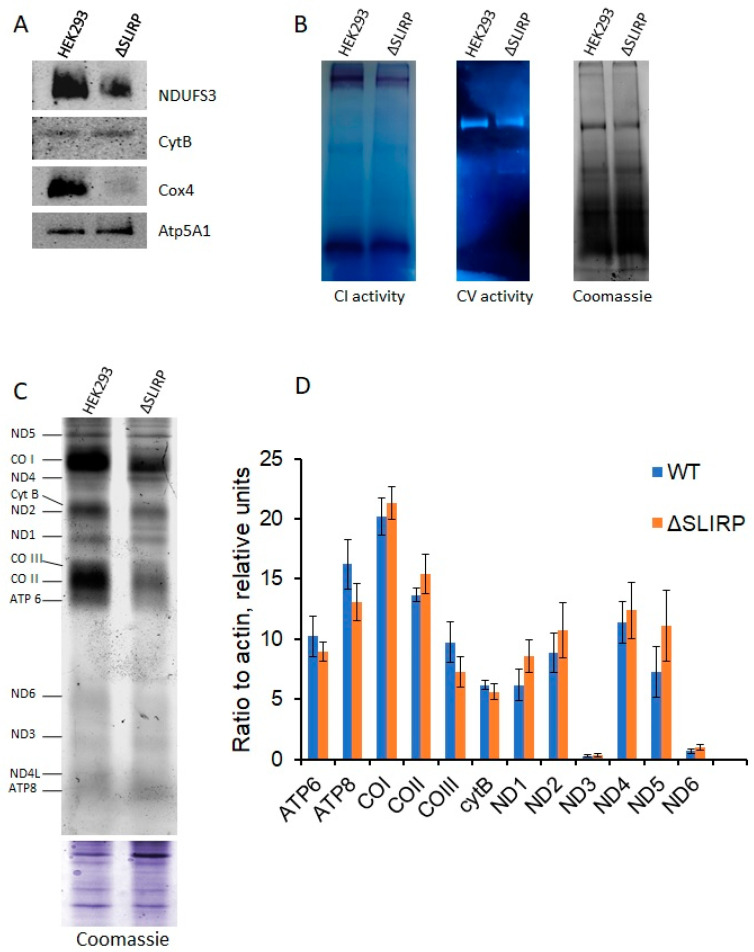
The absence of SLIRP protein results in a decrease in the amounts of complexes I and IV and translation of its mitochondrially encoded subunits. (**A**) Western blot analysis for OXPHOS complexes after Blue Native PAGE of mitochondrial lysates obtained from wild-type and knockout cells. (**B**) In-gel activities of complexes I and V after Blue Native PAGE. (**C**) Analysis of mitochondrial translation products in wild-type and SLIRP knockout cells. Approximately 2 × 10^6^ cells (T-75 flask with 80–90% confluency) were washed with PBS and pre-starved with methionine-free DMEM supplemented with 10% dialyzed FBS for 30 min. Cytosolic translation was blocked by adding cycloheximide up to 200 μg/mL and incubating for 5 min. Subsequently, HPG was added to the medium up to 50 μM, and cells were cultivated for 2 h. Incorporation of HPG was stopped by adding full DMEM supplemented with 10% FBS. The cells were then washed twice with PBS and collected. Mitochondria-enriched fractions were obtained as described in the Materials and Methods section. They were then lysed with SDS, and click chemistry reactions of AF488 azide coupling were performed. Then, proteins were separated using 18% SDS-PAGE. The gels were scanned using the Typhoon^TM^ laser-scanner platform for AF488 fluorescence, and the resulting images were processed using ImageJ software (version 1.54f 29). Further details can be found in the Materials and Methods section. (**D**) Diagram of RT-qPCR of mitochondrial RNAs isolated from wild-type and knockout cells data. Three biological replicates (N = 3) starting from thawing cells from the frozen stock stored in liquid nitrogen were made. Each biological replicate was analyzed in three technical replicates (n = 3). Mean values between the three biological replicates were plotted on the diagram. The error bars represent standard deviations (SD) for three biological replicates. The SD values within each technical replicate were less than 1%. The comparison of the values calculated for each individual mRNA in a parametric *t*-test was performed. In each case, *p*-values were more than 0.05, so the difference between WT and ΔSLIRP samples was not significant.

Next, we determined the quantity of respiratory chain complexes in cells with a deletion in the *SLIRP* gene. To accomplish this, we isolated mitochondria from knockout and wild-type cells and solubilized them using dodecylmaltoside. Subsequently, we conducted Blue-Native electrophoresis followed by Western blot analysis utilizing antibodies against the various complex components. Based on our experimental findings, deletion of the *SLIRP* gene causes a significant reduction in the quantity of complex IV, validating the data obtained on oxygen uptake rate in the presence of FCCP. Furthermore, we observed a significant decrease in the abundance of complex I while no decline was observed in the levels of complex III and V (Figure 2A).

Analysis of the activity of the complexes after blue-native PAGE of digitonin-solubilized mitochondria confirmed a decrease in the enzymatic activity of complex I up to 2 times (N = 3, mean wild type to knockout ratio = 1.85 +/− 0.32), while the activity of complex V in cells with a deletion in the *SLIRP* gene was at the level of wild-type cells (N = 3, mean wild type to knockout ratio = 1.04 +/− 0.14) (Figure 2B).

The reduction in the quantity and activity of complexes I and IV may be due to a change in the efficiency of translation of their mitochondrially encoded subunits. To explore the impact of SLIRP protein absence on mitochondrial protein biosynthesis, we analyzed the mitochondrial translation profiles of knockout and wild-type cells. To accomplish this, cytosolic translation was blocked with cycloheximide, and cells were incubated with L-homopropargylglycine (HPG), which is incorporated into the nascent polypeptide instead of methionine. The incorporated HPG was then cross-linked with AF488 fluorescent dye via click-chemistry reaction for subsequent imaging. Our experimental data demonstrate that deletion in the *SLIRP* gene selectively impacts the synthesis of mitochondrial proteins. Thus, it was observed that the knockout cells demonstrated a decrease in synthesis of complex IV components (CO I, CO II and CO III), while no decrease or a much lesser decrease in the synthesis of other proteins like ND5 or ATP6 was noted (Figure 2C). These observations are consistent with the outcomes of the Western blot analysis. A notable reduction in the quantities of CO II and CO III proteins was observed in SLIRP knockout cells, but cyt b levels remained unchanged compared to wild-type cells (Figure 1D).

A potential cause for the decline in a specific protein’s quantity within mitochondria is linked to the stability of its corresponding mRNA. Some published data suggest that SLIRP impacts the stability of mitochondrial transcripts: siRNA-mediated knockdown of SLIRP leads to a significant decrease in almost all mitochondrial transcripts [16]. By employing RT-qPCR, we examined mitochondrial transcript levels between knockout and wild-type cells, but no notable difference was observed (Figure 2D). Moreover, the selective effect of *SLIRP* gene deletion on individual states of the oxidative phosphorylation chain complex that we observed does not correlate with the possible instability of almost all mRNAs. From our observations, we have suggested that the SLIRP protein may be involved in the regulation of the translation of certain mitochondrial mRNAs.

### 2.3. SLIRP Co-Sediments Together with Small Subunits of Mitochondrial Ribosome

**Figure 3:**
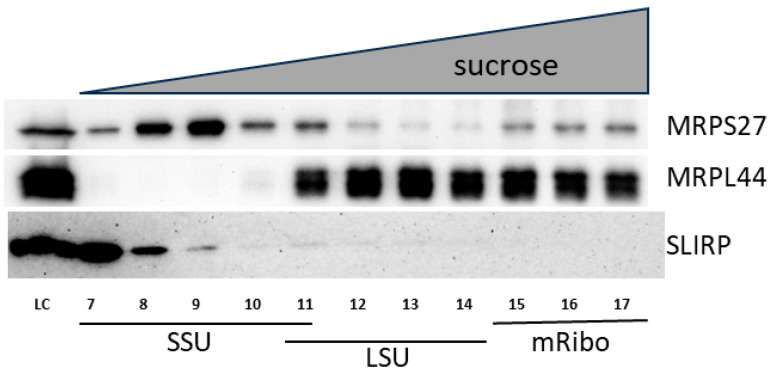
SLIRP co-sediments together with small subunits of mitochondrial ribosomes. HEK293T cell lysates were applied to 10–40% linear sucrose gradients and ultracentrifuged at 80,000× *g* for 18 h, SW-40Ti rotor. Gradients were fractionated from top to bottom (20 fractions, ~600 μL each), proteins from fractions 7–17 were precipitated with methanol-chloroform and applied to Western blot analysis. LC—loading control (0.1%), SSU—fractions of small subunits, LSU—fractions of large subunits, mRibo—associated mitoribosomes.

The initiation stage is crucial for regulating the translation process. However, several significant aspects concerning mammalian mitochondrial translation remain unknown, including how initiation begins on leaderless mRNAs and which entity carries out the initiation, the small ribosomal subunit, or the 55S monosome. We tested the association of the SLIRP protein with the mitochondrial ribosome. To achieve this, HEK293T cell lysates were ultracentrifuged in a sucrose gradient, the gradients were fractionated from top to bottom, and the resulting fractions were analyzed by Western blot analysis. The experiment showed that SLIRP was solely found in fractions corresponding to small ribosomal subunits (Figure 3).

The position of SLIRP in the sucrose gradient can be explained by its involvement in the assembly of small subunits of the mitochondrial ribosome. We conducted ultracentrifugation of lysates from knockout and wild-type cells on sucrose gradients, followed by the analysis of subunit distribution, to verify this hypothesis. Nevertheless, we did not detect any significant variations in subunit distribution, which suggests the absence of differences in mitochondrial ribosome assembly in knockout and wild-type cells (Figure S3).

SLIRP contains an RNA binding motif (RRM) and is also capable of binding SRA RNA [15]. We therefore hypothesized that the SLIRP protein may directly bind some mitochondrial mRNAs and recruit them to the small subunit of the mitochondrial ribosome. In order to test this hypothesis, a number of experiments were carried out on the interaction of recombinant SLIRP with CO II and CYTB mRNA using the EMSA technique, but no direct interaction between recombinant SLIRP and mRNA synthesized in vitro could be detected under our experimental conditions. SLIRP has been identified to create a stable complex with LRPPRC, which is a pentatricopeptide protein. This complex has the ability to bind to various RNAs and to affect their conformation, thus acting as a “global” chaperone [13]. It is likely that interaction with CO II and CYTB mRNA requires the formation of that complex.

## 3. Discussion

Our study discovered that the dysfunction of SLIRP in human HEK293T cells results in impaired mitochondrial respiration due to a deficiency of complex IV and a decrease in the activity and amount of complex I. These findings are consistent with clinical studies that have shown mutations in the *SLIRP* gene lead to mitochondrial encephalomyopathy accompanied by a decrease in quantities of the same complexes [17]. We propose a new method for visualizing newly synthesized mitochondrial polypeptides using click chemistry to analyze mitochondrial translation. This method is significantly cheaper, faster, and safer than the commonly used 35S-methionine labeling. The analysis of the mitochondrial translation profile in cells with a deletion in the *SLIRP* gene revealed that the absence of SLIRP does not result in a general decrease in protein synthesis. However, it has a specific effect on the synthesis of predominantly subunits of complex IV, such as CO I, CO II and CO III.

Cryoelectron microscopy data of mitochondrial ribosomes, accompanied by data from mass spectrometric analysis of the sample under study, suggest that SLIRP may play a role in regulating mitochondrial translation. At the entry site of mRNA into the mitochondrial ribosome, a density similar to the SLIRP–LRPPRC complex was detected. This suggests that the SLIRP–LRPPRC complex mediates the delivery and positioning of mRNA on the mitochondrial ribosome by contacting the mitoribosomal protein PTCD3 [18]. However, our analysis of SLIRP distribution among mitochondrial ribosome fractions revealed that SLIRP co-migrates with small ribosomal subunits and is absent in fractions corresponding to associated mitoribosomes. The data are not controversial regarding the ability of SLIRP to complex with LRPPRC and interact with PTCD3 but indicate its involvement in the translation initiation phase.

SLIRP is considered an RNA-binding protein due to its two RRM domains and ability to bind specific types of RNA in the nucleus [15]. However, in experiments testing the retention of mitochondrial mRNAs in the gel, SLIRP did not exhibit any affinity for RNA, which is consistent with the CLIP analysis data [13]. This may be due to the absence of the MTS motif in the mitochondrial fraction of SLIRP, which is necessary for RNA binding [15]. The SLIRP–LRPPRC complex can relax RNA secondary structures [13]. This is important for 55S initiation in mitochondrial translation, as the efficiency of translation initiation on leaderless mRNAs likely correlates with start codon availability [9]. However, this cannot account for the selective impact of SLIRP depletion on the mitochondrial translation profile we observed. Furthermore, although the 55S monosome has been shown to bind mRNA more efficiently than the 28S in vitro [4], it is unable to translate it in vitro in the presence of the full set of essential translational factors [5]. In bacteria, leaderless mRNAs also exhibit a stronger affinity for the 70S ribosome than for the 30S subunit, but the addition of initiation factors levels off the difference [19]. SLIRP could be a candidate to act as an additional factor that modulates the affinity of the 28S subunit to mRNA, as the functions of mitochondrial initiation factors have diverged from bacterial ones.

## 4. Materials and Methods

### 4.1. Cell Lines, Media, and Growth Conditions

Human cell line HEK293T (ATCC: CRL-3216) and its derivatives were cultured in DMEM (Gibco, Thermofisher Scientific, Waltham, MA, USA) with 4.5 g/L glucose, Glutamax (Gibco, Thermofisher Scientific, Waltham, MA, USA), sodium pyruvate, and 10% (*v*/*v*) FBS (Sigma-Aldrich, Saint-Louis, MO, USA) under a 5% CO_2_ humidified atmosphere at 37 °C. To prevent contamination, DMEM was supplied with 1000 units/mL of penicillin, 100 µg/mL of streptomycin, and 2.5 µg/mL of Amphotericin B.

### 4.2. Generation of Knockout Cell Line

The generation of knockout cell lines with the CRISPR/Cas9 technology was performed as previously described in [20]. sgRNA design was performed using the CRISPOR online platform (http://crispor.tefor.net, accessed 14 December 2022) [21]. The selected sgRNA spacer cDNA sequences (5′-CACCGCGTAGAAGTATCAATCAGC-3′ and 5′-AAACGCTGATTGATACTTCTACGC-3′ for sgRNA1 and 5′-CACCGCACTCGACGCCGCAGTCCA-3′ and 5′-AAACTGGACTGCGGCGTCGAGTGC-3′ for sgRNA2) were synthesized (Evrogen, Moscow, Russia), annealed and inserted between BbsI sites in the pU6-gRNA vector containing the sgRNA scaffold (kindly provided by Dr. Boris Skryabin, University of Munster). HEK293T cells were co-transfected with sgRNAs-pU6 vectors targeting SLIRP exon 1 together with an Cas9/EGFP-containing plasmid using Lipofectamine 3000 reagent (Thermofisher Scientific, Waltham, MA, USA). Then, 24 h after transfection, cells were sorted using FACS Aria SORP (Beckton Dickinson Biosciences, Franklin Lakes, NJ, USA) and screened for individual clones. The gene editing was confirmed by PCR with specific primers (5′-CGGAAGCAGATTCTCTCGTG-3′ and 5′-CTTTGAGGATCACTTGGACC-3′) followed by Sanger sequencing of resulting products.

For the transient expression of SLIRP in knockout cells, the total RNA was extracted from HEK293T cells with TRIzol (ThermoFisher Scientific, Waltham, MA, USA). cDNA was made with a MMLV RT kit (Evrogen, Moscow, Russia). The cDNA copy of SLIRP ORF was amplified by PCR with the primers 5′-gtcaGGATCCATGGCGGCCTCAGC-3′ and 5′-gtcaCTCGAGTCAAAAATCTTTCTTTTCATCATCAGATGT-3′. Plasmid pcDNA5/FRT/TO-SLIRP was constructed by insertion of the cDNA copy of the corresponding ORF between BamHI and XhoI sites. Cloned SLIRP cDNA was Sanger sequenced for the absence of mutations.

### 4.3. Seahorse XF HS MINI Analysis

The oxygen consumption rate (OCR) assay was performed using an Agilent seahorse XFp cell mito stress test (Agilent Technologies, Santa Clara, CA, USA) kit following the manufactory protocol. Cells were seeded in XF 24-well microplates (Agilent Technologies, Santa Clara, CA, USA) at a concentration of 6 × 10^4^ cells per well. OCRs were measured in DMEM XF media supplemented 10 mM glucose, 2 mM L-glutamine, and 1 mM sodium pyruvate under basal conditions and in response to 1.5 μM oligomycin, 2 μM FCCP, and 0.5 μM rotenone/antimycin A, using a Seahorse XF HS mini analyzer (Agilent Technologies, Santa Clara, CA, USA). The actual value of OCR was normalized to the total protein content measured by Bradford assay.

### 4.4. Analysis of Mitochondrial Translation Products

Mitochondrial translation assays were performed using nascent protein labeling by click chemistry. Approximately 2 × 10^6^ cells (T-75 flask with 80–90% confluency) were washed with PBS and pre-starved with methionine-free DMEM supplemented with 10% dialyzed FBS for 30 min. Then, cytosolic translation was inhibited by adding cycloheximide (PanReac, Barcelona, Spain) to 200 μg/mL. After 5 min incubation, L-homopropargilglycine (HPG, Lumiprobe, Moscow, Russia) was added to medium up to 50 μM, and cells were cultivated for 2 h. The incorporation of HPG was stopped by adding full DMEM supplemented with 10% FBS; cells were washed twice with PBS and collected.

The mitochondria-enriched fraction was isolated as follows. Cells were resuspended in 850 μL of RSB Hypo buffer (10 mM Tris-HCl pH 7.5, 10 mM NaCl, 1.5 mM NaCl) and incubated on ice for 20 min. Cells were crashed with Dounce homogenizer on ice (50 strokes, pestle B), and 650 μL of 2.5 × MS buffer (12.5 mM Tris-HCl pH 7.5, 525 mM mannitol, 175 mM sucrose, 2.5 mM EDTA) were immediately added to suspension to prevent mitochondria lysis. Suspension was transferred to the clean pre-chilled tube and diluted twice by adding 1.3 mL of 1 × MS buffer (5 mM Tris-HCl, 210 mM mannitol, 70 mM sucrose, 1 mM EDTA). Large particles (unbroken cells, nuclei, etc.) were sedimented by centrifugation at 1500× *g* for 5 min at 4 °C, the supernatant was transferred to the clean pre-chilled tube, and low-speed centrifugation was performed one more time. Mitochondria from the supernatant were collected by centrifugation at 17,000× *g* for 10 min at 4 °C.

Mitochondria were resuspended in a minimal volume (30–50 μL) of 50 mM Tris-HCl pH 8.1, and the protein concentration was measured by Bradford assay. Mitochondria were lysed by adding 10% SDS up to 1% final concentration and 10 min incubation at room temperature. The 75–100 μL click reaction was carried out in 1× click buffer (Lumiprobe, Moscow, Russia), 20–40 ug of mitochondrial proteins, 0.1 mM AF 488 azide (10 mM stock solution in DMSO, Lumiprobe, Moscow, Russia) and 1 mM ascorbic acid (Lumiprobe, Moscow, Russia). After mixing the reagents, the reaction tube was filled with nitrogen and incubated for 8–16 h at room temperature. After completing the reaction, the unbound fluorophore was removed by size-exclusion chromatography at a Micro Bio-Spin^TM^ column (BioRad, Hercules, CA, USA) pre-equilibrated with PBS.

Eluted proteins were precipitated with methanol–chloroform, dissolved in 20–30 μL of 1xLaemmli loading buffer and applied on 18% PAGE. Mitochondrial translation products were visualized with the Typhoon^TM^ laser-scanner platform (Amersham, Amersham, UK) and assessed with ImageJ software (version 1.54f 29 June 2023, NIH, Bethesda, MD, USA).

### 4.5. Western Blot

Western blot analyses were performed conventionally using the following rabbit antibodies: anti-SLIRP (26006-1-AP, Proteintech, Manchester, UK), anti-MRPS27 (17280-1-AP, Proteintech, Manchester, UK), anti-MRPL44 (16394-1-AP, Proteintech, Manchester, UK), anti-Cox4 (11242-1-AP, Proteintech, Manchester, UK), anti-COII (55070-1-AP, Proteintech, Manchester, UK), anti-COIII (55082-1-AP, Proteintech, Manchester, UK), anti-CytB (55090-1-AP, Proteintech, Manchester, UK), anti-NDUFS3 (15066-1-AP, Proteintech, Manchester, UK), anti-Atp5A1 (14676-1-AP, Proteintech, Manchester, UK), and ECL Anti-rabbit IgG HRP linked (NA934V, GE Healthcare, MA, USA), according to the manufacturer’s protocols. Blots were stained with SuperSignal West Pico ECL reagents (ThermoFisher Scientific, Waltham, MA, USA) and visualized with ChemiDoc imaging system (Bio-Rad, CA, USA).

### 4.6. Blue-Native Electrophoresis

BN-PAGE was performed as described in [22]. Briefly, 100 μg of isolated mitochondria was resuspended in 10 μL of solubilization buffer A (50 mM NaCl, 2 mM 6-aminohexanoic acid, 1 mM EDTA, 50 mM imidazole-HCl, pH 7) and 3 μL of 20% digitonin or 1.5 μL of 20% dodecylmaltoside. The mitochondria were solubilized 20 min on ice, and debris was removed by 1 h centrifugation at 4 °C and 25,000× *g*. Coomassie dye (5% Coomassie Blue G-250 dye stock solution, suspended 500 mM 6-aminohexanoic acid) was added to the supernatant to give a detergent/dye ratio of 8, and the samples were subjected to electrophoresis in a 4–14% gradient gel. After electrophoresis, either in-gel respiratory complex activities were measured, or Western blot analysis was performed. Measurement of the complex I and V activities of the gel was performed according to [23]. For complex I activity assays, gels were incubated in solution containing 0.14 mM NADH, 1 mg/mL Nitro Blue tetrazolium (NBT) and 0.1 M Tris HCl, pH 7.4, until the appearance of violet bands. The reaction was stopped with 10% acetic acid solution. For complex V activity assays, gels were washed several times in deionized water and incubated for 16 h in 35 mM Tris, 270 mM glycine, 14 mM MgSO_4_, 5 mM ATP, and 0.2% (*w*/*v*) Pb(NO_3_)_2_. The reaction was stopped with 50% methanol. All enzymatic assays were completed in 3 biological replicates. The gels after enzymatic assays were pictured and scanned in a ChemiDoc Imaging System (Bio-Rad, Hercules, CA, USA), and quantification was performed using ImageJ software (version 1.54f 29, NIH, Bethesda, MD, USA). The data were expressed as mean ratios of wild-type to knockout values +/− SD.

### 4.7. Quantitatively RT-PCR

Total RNA was extracted from the WT and SLIRP knockout cells with TRIzol (ThermoFisher Scientific, Waltham, MA, USA). cDNA was made with a MMLV RT kit (Evrogen, Moscow, Russia). These cDNAs were amplified by qPCR using SsoAdvanced Universal SYBR Green Supermix (Bio-Rad, Hercules, CA, USA) on a CFX96 Touch Real-Time PCR Detection System (BioRad, Hercules, CA, USA) with a set of primer pairs designed for each individual mitochondrial mRNA and actin mRNA as an internal calibrator. The sequences of the primers used are given in Table S1. The values of threshold cycles (Ct) were taken from CFX Maestro 2.0 software (version 5.0.021.0616). ΔCt ratios to actin were calculated for each mRNA in Microsoft Excel using the following equation:

$${Ratio(mRNA)=2}^{-[Ct\left(mRNA\right)-Ct\left(actin\right)]}$$

These ratios to actin were calculated for all indicated mRNAs in the WT and SLIRP knockout cells. Three biological replicates (N = 3) starting from thawing cells from the frozen stock stored in liquid nitrogen were made. Each biological replicate was analyzed in three technical replicates (N = 3). Mean values between the three biological replicates were plotted on the diagram. The error bars represent standard deviations (SD) calculated in Microsoft Excel for three biological replicates. The comparison of the values calculated for each individual mRNA in parametric *t*-test was made in R studio.

### 4.8. Fractionation of Mitochondrial Ribosomes

Approximately 10^7^ cells were collected, washed twice with PBS, and lysed in 1 mL of 10 mM HEPES pH 7.4, 120 mM KCl, 20 mM magnesium acetate, 5 mM β-mercaptoethanol, 1.7% Triton X-100 for 30 min on ice. After clarification at 25,000× *g* for 30 min, lysates were applied to a 10–40% linear sucrose gradient prepared on the same buffer without detergent. After ultracentrifugation at 80,000× *g* for 18 h (SW-40Ti rotor, Beckman Optima XE-90 centrifuge), gradients were fractionated with Gradient Station ip (Biocomp Instruments, Fredericton, NB, Canada). Proteins in fractions were sedimented with methanol/chloroform and applied to Western blotting.

## 5. Conclusions

Here we have shown that deletion of the *SLIRP* gene, resulting in the absence of this protein, leads to a decrease in the biosynthesis of complex IV subunits encoded in the mitochondrial genome. This leads to loss of complex IV activity and significant impairment of mitochondrial ATP synthesis, although ATP synthase activity is not affected. We also found that SLIRP co-sediment with small mitoribosomal subunits in sucrose density gradients, suggesting that SLIRP may be a translational initiation regulator of some mitochondrial mRNAs. 

## Data Availability

Data is contained within the article.

## Supplementary Materials

The following supporting information can be downloaded at https://www.mdpi.com/article/10.3390/ijms25010093/s1.
