# Supplementary material for: Mitochondrial Protein SLIRP Affects Biosynthesis of Cytochrome c Oxidase Subunits in HEK293T Cells
Source: Int J Mol Sci. 2023 Dec 20;25(1):93. doi: 10.3390/ijms25010093 (PMC10779364; doi:10.3390/ijms25010093)
Supplement: Supplementary file 1 [file ijms-25-00093-s001.zip › Figure S1.pdf]

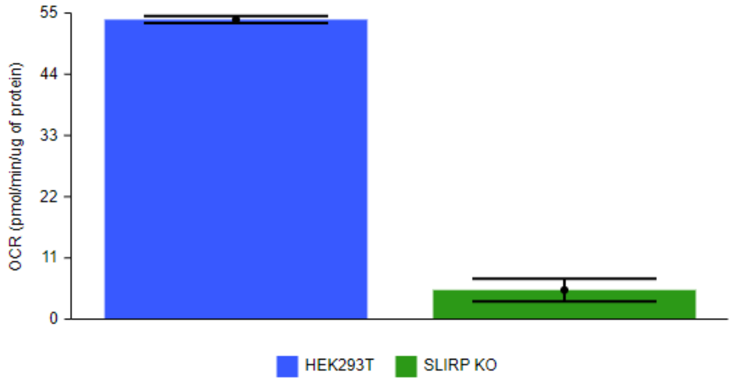

Figure S1. ATP production coupled with respiration. Coupled ATP production rates of HEK293T and SLIRP KO were measured using Seahorse FX Mini bioanalyzer. The values are expressed as differences of oxygen consumption rates (OCR) at basal conditions and in the presence of ATP-synthase inhibitor oligomycin. All data were normalized to total protein quantity measured by Bradford assay after OCR measurements. Resulting diagram represents mean values of 3 biological replicates +/-SD.
