# Supplementary material for: Mitochondrial Protein SLIRP Affects Biosynthesis of Cytochrome c Oxidase Subunits in HEK293T Cells
Source: Int J Mol Sci. 2023 Dec 20;25(1):93. doi: 10.3390/ijms25010093 (PMC10779364; doi:10.3390/ijms25010093)
Supplement: Supplementary file 1 [file ijms-25-00093-s001.zip › Figure S2.pdf]

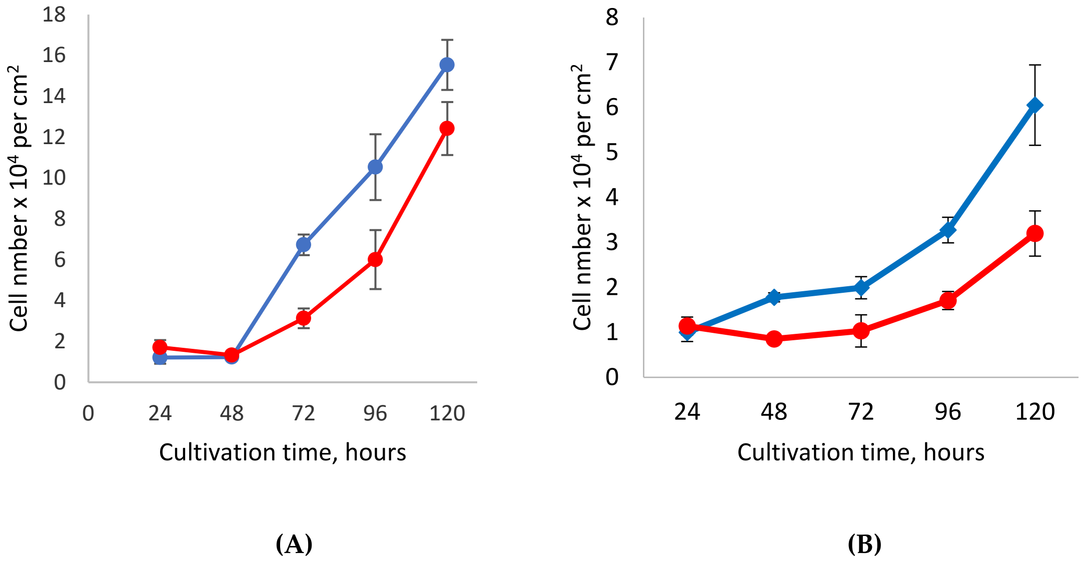

Figure S2. Analysis of proliferative activity of HEK293T and SLIRP knock-out cells. The series of approximately 2.5 x 10^4 cells were placed in a well of a 24 well plates (three for each line) and cultivated in DMEM high glucose (4.5 g/L) (Gibco, Thermo Scientific, USA) supplemented with 10% FBS (Corning, USA), 4 mM glutamine, 1 mM sodium pyruvate and 100 U/ml penicillin-streptomycin (Thermo Scientific, USA), or DMEM galactose medium (Paneco, Russia) with the same additives. Viable cell density was determined by trypan blue exclusion method and counting using an automated TC-20 cell counter (Bio-Rad, USA) every 24 hours. A – cultivation in high glucose medium; B – cultivation in galactose medium. Blue line – HEK293T cells, Red line – SLIRP knock-out cells. Mean data of 3 biological replicates +/- SD presented.
