# Supplementary material for: Mitochondrial Protein SLIRP Affects Biosynthesis of Cytochrome c Oxidase Subunits in HEK293T Cells
Source: Int J Mol Sci. 2023 Dec 20;25(1):93. doi: 10.3390/ijms25010093 (PMC10779364; doi:10.3390/ijms25010093)
Supplement: Supplementary file 1 [file ijms-25-00093-s001.zip › Figure S3.pdf]

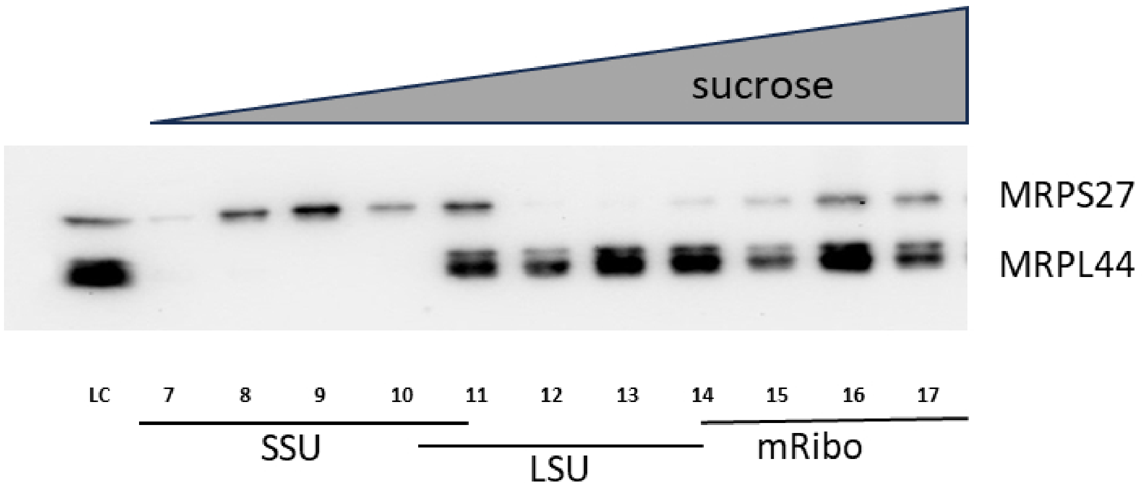

Figure S3. Deletion in SLIRP gene does not affect mitochondrial ribosome assembly. Knockout cells were lysed, lysate was applied to 10% - 40% sucrose gradient and ultracentrifuged. After centrifugation gradient was fractionated from top to bottom, and proteins from each fraction were precipitated and applied to western blot analysis with antibodies to MRPS27 and MRPL44 proteins together. LC – loading control, SSU – small mitoribosomal subunits, LSU – large mitoribosomal subunits, mRibo – associated mitoribosoms.
