# Supplementary material for: Mitochondrial Protein SLIRP Affects Biosynthesis of Cytochrome c Oxidase Subunits in HEK293T Cells
Source: Int J Mol Sci. 2023 Dec 20;25(1):93. doi: 10.3390/ijms25010093 (PMC10779364; doi:10.3390/ijms25010093)
Supplement: Supplementary file 1 [file ijms-25-00093-s001.zip › Table S1.pdf]

Table S1. Oligonucleotides used in RT-qPCR analysis.

Name	Sequence 5′ – 3′
qATP6_F	CCATACACAACACTAAAGGACG
qATP6_R	GTGTAGGTGTGCCTTGTGG
qATP8_F	CCACCATAATTACCCCCATACTCC
qATP8_R	ATGGGCTTTGGTGAGGG
qCOX1_F	GGCACAGCTCTAAGCCTCC
qCOX1_R	CTCCGGCCTCCACTATAGC
qCOX2_F	CTACAAGACGCTACTTCCCC
qCOX2_R	CGATGAGGACTAGGATGATGGC
qCOX3_F	GCCCTCTCAGCCCTCC
qCOX3_R	GGCTCAGAAAAATCCTGCG
qCYTB_F	AAAATTAATTAACCACTCATTCATCGACC
qCYTB_R	CCGATGTGTAGGAAGAGGCAG
qND1_F	CCACCTCTAGCCTAGCCGTTTA
qND1_R	GGGTCATGATGGCAGGAGTAAT
qND2_F	CCAACCCGTCATCTACTCTACC
qND2_R	TGATGAGTATTGATTGGTAGTATTGG
qND3_F	ACACCCTCCTAGCCTTACTAC
qND3_R	GGATGACATAACTATTAGTGGCAGG
qND4_F	TATTACTACCACTGACATGACTTTCC
qND4_R	TTGTAGGGAGATTAGTATAGAGAGGTAGAG
qND5_F	CCTTACCACCCTCGTTAACCC
qND5_R	CACAGTGAGAATTCTATGATGGACC
qND6_F	TTGTTTCTGTTGAGTGTGGGTTTAG
qND6_R	CCCCCATGCCTCAGGATAC
qRNR1_F	TAGAGGAGCCTGTTCTGTAATCGAT
qRNR1_R	CGACCCTTAAGTTTCATAAGGGCTA
qRNR2_F	GGACTAACCCCTATACCTTCTGC
qRNR2_R	TTTAGAGGGTTCTGTGGGC
qACTIN_F	CACCATTGGCAATGAGCGGTTC
qACTIN_R	AGGTCTTTGCGGATGTCCACGT
qGAPDH_F	GTCTCCTCTGACTTCAACAGCG
qGAPDH_R	ACCACCCTGTTGCTGTAGCCAA
